# Real-world impact of primary immune thrombocytopenia and treatment with thrombopoietin receptor agonists on quality of life based on patient-reported experience: Results from a questionnaire conducted in Switzerland, Austria, and Belgium

**DOI:** 10.1371/journal.pone.0267342

**Published:** 2022-04-21

**Authors:** Alicia Rovó, Nathan Cantoni, Kaveh Samii, Axel Rüfer, Giedre Koenen, Sandra Ivic, Davide Cavanna, Rudolf Benz

**Affiliations:** 1 INSELSPITAL, Department of Haematology and Central Haematology Laboratory, Bern University Hospital, Bern, Switzerland; 2 Division of Oncology, Hematology and Transfusion Medicine, Kantonsspital Aarau, Aarau, Switzerland; 3 Department of Oncology, Division of Hematology, University Hospitals of Geneva, Geneva, Switzerland; 4 Division of Hematology, Cantonal Hospital Lucerne, Lucerne, Switzerland; 5 Novartis Pharma Schweiz AG, Risch-Rotkreuz, Switzerland; 6 Division of Hematology and Oncology, Cantonal Hospital, Münsterlingen, Switzerland; Qatar University, QATAR

## Abstract

**Aims of the study:**

Thrombopoietin receptor agonists (TPO-RAs) are approved for immune thrombocytopenia (ITP), but their impact on health-related quality of life (HRQoL) remains poorly investigated in clinical practice. This observational study aimed to gain insight into real-world patient-reported experiences of the burden of ITP and TPO-RAs.

**Method:**

An online questionnaire of closed questions was used to collect views of patients with primary ITP from Switzerland, Austria, and Belgium, between September 2018 and April 2020.

**Results:**

Of 46 patients who completed the questionnaire (total cohort), 41% were receiving TPO-RAs. A numerically higher proportion of patients reported being free from symptoms at the time of the questionnaire (54%) than at diagnosis (24%), irrespective of treatment type. Bleeding, the most frequently reported symptom at diagnosis (59%), was reduced at the time of the questionnaire (7%). Conversely, fatigue was reported by approximately 40% of patients at both diagnosis and the time of the questionnaire. Having a normal life and their disease under control was reported by 83% and 76%, respectively, but 41% were worried/anxious about their condition. Nearly 50% reported that ITP impaired their engagement in hobbies/sport or energy levels and 63% reported no impact on employment. When stratified by TPO-RA use, bleeding was better controlled in those receiving TPO-RAs than not (0% vs 11%). A numerically lower proportion receiving TPO-RAs than not reported worry/anxiety about their condition (16% vs 59%) and shifting from full-time to part-time employment (11% vs 22%). Similar proportions were satisfied with their therapy whether they were receiving TPO-RAs or not (89% vs 85%).

**Conclusions:**

Many factors affect HRQoL in patients with ITP. Of patients receiving TPO-RAs, none experienced bleeding at the time of the questionnaire; they also showed a more positive perspective for some outcomes than those not using TPO-RAs. However, fatigue was not reduced by any treatment.

## Introduction

Immune thrombocytopenia (ITP) is an autoimmune disorder characterized by low platelet count (peripheral blood < 100 × 10^9^/L), as a result of platelet destruction and/or impaired production [1, 2]. ITP affects people of all ages, but its incidence peaks in children and young adults as well as the elderly [3]. In Europe, its incidence in adults ranges from 1 to 4 per 100 000 population [4, 5]. Owing to the chronic nature of adult ITP, cases accumulate over time, and the age- and sex-adjusted prevalence ranges from 1 to 5 per 10 000 population [6–8]. According to the European Medicines Agency definition, ITP is a rare disease (prevalence < 5 per 10 000 people) [8, 9].

Symptoms of ITP are variable and some affected individuals have no symptoms [10, 11]. Symptomatic patients may experience bruising or spontaneous bleeding and are at risk of serious, potentially fatal bleeding events [2, 10]. Patients also report petechiae/purpura (rash of blood spots on the skin), insomnia, and depression, and many report fatigue [12, 13].

There are no tests that can be used to definitively diagnose ITP [2, 10, 14]. After the exclusion of other diseases that can lead to low platelet counts (e.g., autoimmune disease or lymphoma), patients for whom there is no underlying cause of thrombocytopenia are classified as having primary ITP (accounts for 80% of patients with ITP) [2, 15]. Those in whom low platelet count is attributed to other causes are classified as having secondary ITP [2, 15]. Patients can also be classified according to duration of disease from diagnosis as being newly diagnosed (< 3 months), having persistent ITP (3–12 months), or having chronic ITP (> 12 months), the latter of which accounts for the majority of cases in adults [1, 16].

ITP can have an impact on patients’ lives for many reasons: symptoms such as bleeding and fear of bleeding, non-bleeding symptoms such as fatigue, and worry about their condition all have a negative impact on patients’ health-related quality of life (HRQoL) [17–23]. In line with this, studies have demonstrated that patients with ITP have worse HRQoL than age- and sex-matched comparisons or the general population, and comparable HRQoL to patients with other chronic conditions [23–26].

The goal of treatment in ITP is to maintain platelet levels, with minimal toxicity, in order to reduce bleeding and prevent severe bleeding [13, 14]. Several treatment options are available for patients with ITP [14]. Corticosteroids are the standard initial therapy for newly diagnosed patients and also for patients with relapsed disease, whereas patients with persistent or chronic disease will require subsequent-line therapies. Treatments that patients receive to manage their condition can also affect their lives, either positively in those who respond to treatment, or negatively in those who do not respond to treatment or who experience side effects [20–22, 26–30]. Corticosteroids in particular are associated with bothersome side effects, especially when used over a long period of time. They can therefore have a negative impact on HRQoL and the duration over which patients can receive them is limited [14, 23, 26, 30].

In studies of patients with ITP, patients’ perspectives on how their disease and treatments affect their lives are typically assessed using patient-reported outcome instruments, which give a score for HRQoL (including sub-scores for individual domains of life) or the severity of symptoms [5, 17, 21–23, 25, 27, 31–33]. However, the scores derived from these instruments may not give the full picture, because they do not capture patients’ feelings and opinions about their lives or ability to engage in specific daily activities (e.g., sport, work, holiday), or effects on relationships [23, 34]. Furthermore, these instruments are not widely used in clinical practice.

Thrombopoietin receptor agonists (TPO-RAs) are a class of subsequent-line therapy used in patients with ITP that exert therapeutic effects mainly by stimulating platelet production in the bone marrow [14]. Three TPO-RAs have been approved for use in the European Union since 2009, 2010 and 2019, respectively, for patients with ITP who are refractory to initial therapies: [35–37] romiplostim (Nplate^®^; once-weekly subcutaneous injection; Amgen, USA) for patients with primary ITP, eltrombopag (Revolade™; once-daily oral; Novartis, Switzerland) for patients with persistent/chronic ITP, and avatrombopag (Doptelet^®^; once-daily oral; Swedish Orphan Biovitrum AB, Sweden) for patients with primary chronic ITP.

As part of randomized controlled trials (RCTs) and extension studies, TPO-RAs have been shown to improve HRQoL versus placebo or standard of care [25, 26, 28]. One real-world study that investigated HRQoL in patients with chronic ITP who switched to TPO-RAs (including patients who switched from one TPO-RA to another) also demonstrated significantly better outcomes for energy/fatigue and emotional wellbeing compared with the period before switching [32]. However, despite the growing use of TPO-RAs in clinical practice and the fact that they can be received long term, their impact on patients’ lives and HRQoL in the real world has not been extensively investigated [26].

A recent international real-world study, the ITP World Impact Survey (I-WISh), used an online survey to assess the impact of ITP on HRQoL, emotional health, work productivity, and need for caregivers in 1400 patients across 13 countries, and to compare physicians’ and patients’ perceptions of the disease [38, 39]. The I-WISh indicated the multifaceted impact that ITP has on patients’ lives.

The aim of this real-world study was to gain insight into the views and feelings of a group of patients with primary ITP across Switzerland, Austria, and Belgium about their symptoms and the impact of the disease on their daily lives. Because currently available HRQoL questionnaires do not fully capture patients’ thoughts, fears, and treatment satisfaction in a single instrument, an online questionnaire was developed and used.

Among patients receiving TPO-RAs, data were also collected on treatment-related burden and compared with those who were not receiving TPO-RAs. At the time of the study, only romiplostim and eltrombopag were approved for use in patients with ITP in Europe.

## Materials and methods

### Study design

This was a real-world, prospective, cross-sectional online survey-based study that was conducted to gain insight into the views and feelings of participating patients with primary ITP on their symptoms, disease burden, the impact of the disease on their daily lives, and their treatment-related burden. Data were collected using an online questionnaire that was designed specifically for the present study.

### Inclusion criteria and participant recruitment

Patients with primary ITP, who were aged at least 18 years, were eligible for inclusion in this study. Patients were identified from clinical practice by haematology specialists at participating haematology clinics across Switzerland, Austria, and Belgium.

Once healthcare professionals classified a patient as eligible for participation, they could provide them with a sealed envelope that contained an explanation of the study (including the importance of participating and providing accurate responses, and clarification that any responses were anonymous) and a unique access code that patients could use to log in to the online questionnaire if they wished to participate in the study. Access codes could not be linked to the patient completing the questionnaire, or to the physician who provided the envelope, ensuring complete anonymity of the patient and their data. Owing to the small number of patients seen at many of the participating haematology clinics, patient recruitment (e.g. how many patients were recruited from each clinic, how many patients started/completed the questionnaire), was not monitored to further protect patient anonymity.

Patients were recruited and data collected during three active recruitment windows (each of 3 months duration) between September 2018 and April 2020.

### HRQoL questionnaire

Owing to the fact that existing standardized quality of life questionnaires did not explore the range of topics that were identified for investigation in the present study, a new questionnaire was developed. This questionnaire was designed by a scientific steering committee composed of four haematologists from Switzerland with clinical experience in ITP. To ensure that the language in the questionnaire was accessible to and understandable by patients with ITP, the phrasing of questions and terminology used was consistent with that used by haematologists in clinical practice when discussing with their patients about their physical symptoms, emotional wellbeing and quality of life. Furthermore, for some symptoms, images were included alongside the medical terms to help patients recognize what was being discussed, and for other symptoms, clarifying text was included. All members of the scientific steering committee agreed with the content and no additional validation of the questionnaire was performed. Novartis Pharma Schweiz AG played an administrative role in the questionnaire development and did not influence the design of the questionnaire.

The questionnaire comprised five sections of closed questions (screening; details of diagnosis; impact on daily life and emotional burden; impact on work, finances, and use of a caregiver; and treatments; S1 File) and was designed to take approximately 15 minutes to complete. Note that data on use of a caregiver are not shown in the present study because ITP is not associated with need for a caregiver. The questionnaire is available in five languages (English, German, Dutch, Italian, and French). An English version of the questionnaire is provided in S1 File. Surveys in the other languages can be obtained by contacting Novartis (swissonco.medical@novartis.com).

Responses to the questionnaire were collected through multiple-choice questions (participants could select one or more answers), a four-point scale, or a sliding scale. Impact on work productivity over the previous week was assessed using a productivity score, based on a scale of 1 (completely prevented me from working) to 100 (had no effect on my work). Treatment satisfaction was defined as a score of at least 61/100 based on a scale ranging from 1 (not at all satisfied) to 100 (completely satisfied). The threshold used to define treatment satisfaction used the Net Promotor Score^®^ as a guide [40] (converted to a scale of 100), which would have covered scores of at least 71. This was then expanded to include scores of 61–71, because these were still classed as being a positive response. Further details about how responses were collected are included in figure and table legends, where applicable.

### Statistical analysis

Responses to questionnaires were calculated as proportions (n/N; with N representing the total number of participants who responded to the individual question), means, and medians (interquartile range). Data for bleeding symptoms were categorized as cutaneous bleeding (purpura, petechiae, haematoma, prolonged bleeding from cuts), mucosal bleeding (nosebleeds, heavy menstrual bleeding), and internal bleeding (organ bleeding, blood in urine or stool).

Responses were calculated for the total cohort and stratified by TPO-RA usage. The sample size was not pre-determined because all data were reported descriptively and no statistical analysis was performed. The study was not powered to detect statistically significant differences according to TPO-RA use.

### Ethics approval and consent to participate

Owing to the fact that all data were anonymized, the study did not fall within the remit of cantonal or federal law Human Research Act. Although the Ethikkommission Nordwest- und Zentralschweiz (EKNZ; Ethics Committee Northwest and Central Switzerland; eknz@bs.ch) therefore did not have to officially approve the project, the EKNZ reviewed the submitted documents and confirmed that the study fulfilled the general ethical and scientific standards for research in humans.

Participants could choose whether to initiate and complete the questionnaire. At the start of the questionnaire (S1 File), patients needed to provide confirmation that they were over 18 years of age and that they gave permission for their data (without personally identifiable information) to be used in publications, by selecting ‘agree’. If patients did not accept these conditions, they could not complete the rest of the questionnaire and were therefore not included in the study.

## Results

### Demographic and clinical characteristics

Over the study period, 46 patients completed the questionnaire (total cohort). Demographic and clinical characteristics of the total cohort are summarized in Table 1. Most patients who completed the questionnaire were from Switzerland (72% [n = 33/46]), then Belgium (22% [n = 10/46]), and then Austria (7% [n = 3/46]). The median age of the cohort was 50 years (range: 18–80 years), and 52% (n = 24/46) of participants were female. Most patients (85% [n = 39/46]) were currently receiving therapy. The highest proportions of patients in the total cohort reported that they were receiving corticosteroids (46% [n = 21/46]) or TPO-RAs (41% [n = 19/46]).

**Table 1:** Summary of demographic and clinical characteristics of the total cohort and the TPO-RA-stratified cohort.

	Total cohort (N = 46)	Using TPO-RA (n = 19)	Not using TPO-RA (n = 27)
**Sex, n (%)**			
Male	22 (48)	11 (58)	11 (41)
Female	24 (52)	8 (42)	16 (59)
**Country, n (%)**			
Switzerland	33 (72)	14 (74)	19 (70)
Austria	3 (7)	1 (5)	2 (7)
Belgium	10 (22)	4 (21)	6 (22)
Age, years, median (IQR)	50 (32, 59)^†^	51 (34, 59)^‡^	45 (30, 61)^§^
Age at diagnosis, years, median (IQR)	41 (30, 56)	43 (31, 56)	35 (29, 57)
Time since diagnosis, years, median (IQR)	2.5 (0, 9)	3 (1, 10)	2 (0, 9)
Number of physicians seen before diagnosis, median (IQR)	3 (2, 3)	3 (2, 3)	3 (2, 3)
Current health status, median (IQR)^¶^	76.5 (55, 93)	78 (55, 96)	76 (50, 93)
**Treatments currently received, n (%)**			
Steroids	21 (46)	7 (37)	14 (52)
Androgens	1 (2)	0 (0)	1 (4)
Cyclosporin	2 (4)	1 (5)	1 (4)
Rituximab	5 (11)	3 (16)	2 (7)
IVIg	6 (13)	1 (5)	5 (19)
Platelet concentrates	1 (2)	0 (0)	1 (4)
Splenectomy	2 (4)	1 (5)	1 (4)
TPO-RA^#^	19 (41)	19 (100)	0 (0)
Other	8 (17)	2 (11)	6 (22)
Alternative	2 (4)	2 (11)	0 (0)
No therapy	7 (15)	0 (0)	7 (26)

Abbreviations: IQR, interquartile range; IVIg, intravenous immunoglobulin; TPO-RA, thrombopoietin receptor agonist.

^†^Age range 18–80 years.

^‡^Age range 26–73 years.

^§^Age range 18–80 years.

^¶^Based on a scale of 1 (not at all satisfied) to 100 (very satisfied).

^#^At the time of the study, only romiplostim and eltrombopag were approved for use in patients with ITP in Europe.

When stratified by TPO-RA use, there were some numerical differences in baseline characteristics between the cohort using and those not using TPO-RAs for the proportion of female patients (42% [n = 8/19] vs 59% [n = 16/27]), and the median age at diagnosis (43 vs 35 years) and at the time of the questionnaire (51 vs 45 years). There were also some numerical differences between the cohorts for treatments currently received: a numerically lower proportion of patients using than those not using TPO-RAs were receiving steroids (37% [n = 7/19] vs 52% [n = 14/27]) and intravenous immunoglobulin (5% [n = 1/19] vs 19% [n = 5/27]), and a numerically higher proportion of patients using than those not using TPO-RAs were receiving rituximab (16% [n = 3/19] vs 7% [n = 2/27]). Furthermore, 26% (n = 7/27) of patients in the cohort not receiving TPO-RAs were not receiving any other therapy.

### Symptom burden at diagnosis and at the time of the questionnaire

**Fig 1:**
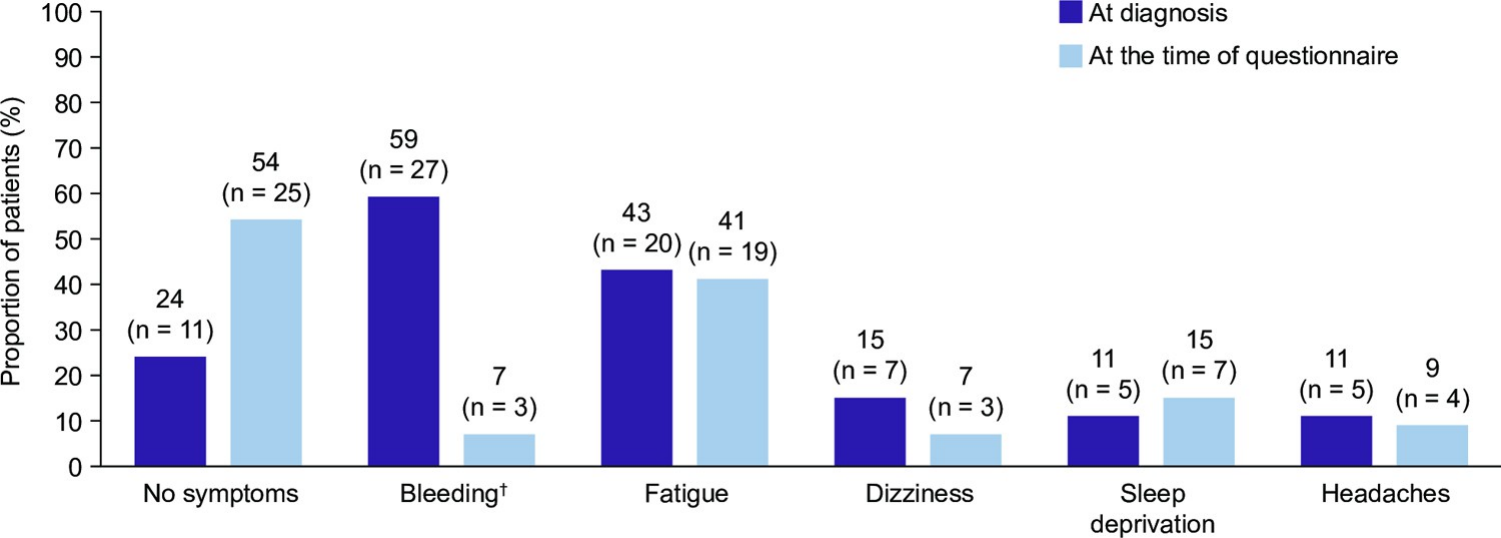
Symptoms at diagnosis and at the time of the questionnaire (total cohort). Data in the figure are presented as the proportion of patients reporting each symptom within the total cohort (N = 46). Patients indicated their symptoms through a multiple-choice question and could select more than one symptom. For patients who indicated they experienced bleeding, they were prompted in a subsequent question to indicate the type of bleeding that they had experienced. ^†^Bleeding included cutaneous bleeding (purpura, petechiae, haematoma, prolonged bleeding from cuts), mucosal bleeding (nosebleeds, heavy menstrual bleeding), and internal bleeding (organ bleeding, blood in urine or stool). Of 27 patients who reported bleeding at diagnosis, 26 reported cutaneous bleeding (96%), 18 reported mucosal bleeding (67%), and 3 reported internal bleeding (11%). Of the 3 patients who reported bleeding at the time of the questionnaire, 3 reported cutaneous bleeding (100%), 2 reported mucosal bleeding (67%), and 1 reported internal bleeding (33%).

A numerically higher proportion of patients in the total cohort reported being free from symptoms at the time of the questionnaire (54% [n = 25/46]) than at diagnosis (24% [n = 11/46]; Fig 1). At diagnosis, bleeding was the symptom reported by the highest proportion of patients (59% [n = 27/46]), but this was reduced at the time of the questionnaire (7% [n = 3/46]). Fatigue was the most commonly reported non-bleeding symptom both at diagnosis (43% [n = 20/46]) and at the time of the questionnaire (41% [n = 19/46]), and it was also the symptom that the highest proportion of patients who had at least one symptom wanted to resolve (33% [n = 4/12]).

**Fig 2:**
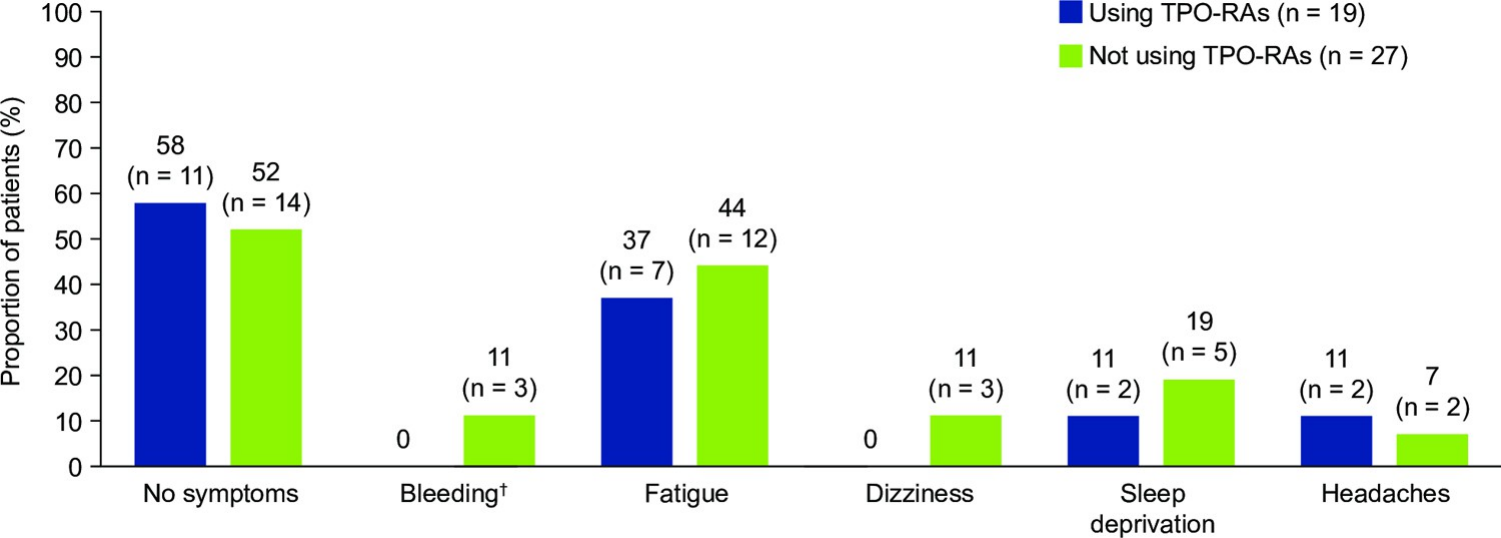
Symptoms at the time of questionnaire according to TPO-RA usage. Data in the figure are presented as the proportion of patients reporting each symptom within the total cohort (N = 46). Patients indicated their symptoms through a multiple-choice question and could select more than one symptom. Patients who responded that they had experienced bleeding were prompted in a subsequent question to indicate the type of bleeding that they had experienced. ^†^Bleeding included cutaneous bleeding (purpura, petechiae, haematoma, prolonged bleeding from cuts), mucosal bleeding (nosebleeds, heavy menstrual bleeding), and internal bleeding (organ bleeding, blood in urine or stool). Of the three patients who reported bleeding in the cohort not using TPO-RAs, three reported cutaneous bleeding (100%), two reported mucosal bleeding (67%), and one reported internal bleeding (33%). Abbreviation: TPO-RA, thrombopoietin receptor agonist.

When stratified by TPO-RA use, data were aligned with the total cohort. Numerically lower proportions of patients receiving than those not receiving TPO-RAs reported bleeding (0% [n = 0/19] vs 11% [n = 3/27), dizziness (0% [n = 0/19] vs 11% [n = 3/27]), and sleep deprivation (11% [n = 2/19] vs 19% [n = 5/27]; Fig 2). Consistent with the total cohort, fatigue (37% [n = 7/19] vs 44% [n = 12/27]) was the symptom reported by the highest proportion of patients whether they were receiving TPO-RAs or not.

### Impact of ITP on patients’ lives and emotions

**Fig 3:**
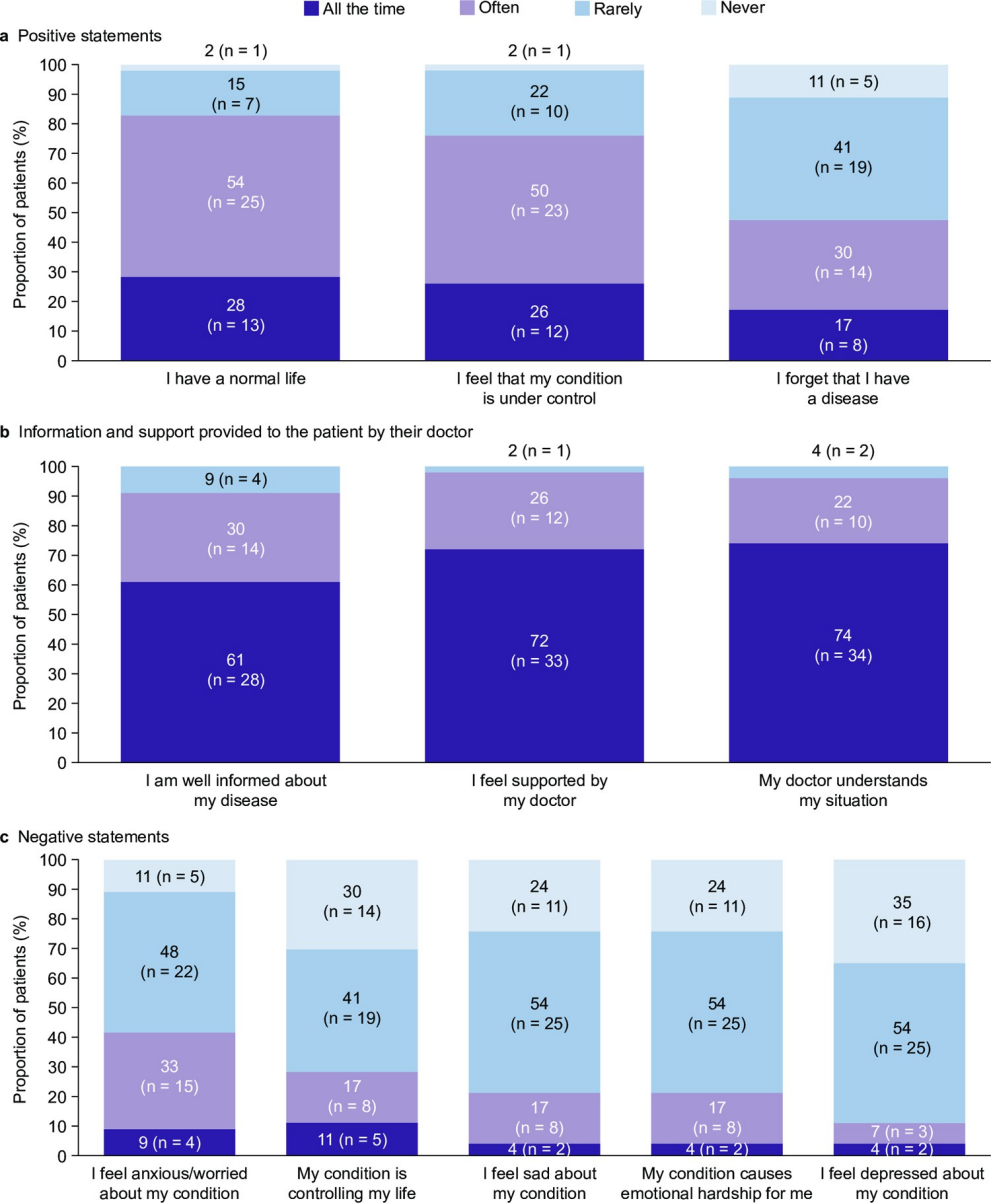
Patients’ feelings about ITP in the total cohort. Patients could select a single response per statement. Data are presented as the proportion of patients per category for each statement (N = 46 for all figures). Abbreviation: ITP, immune thrombocytopenia.

When asked to respond to positive and negative statements about the impact of ITP on their lives and emotions, approximately 80% of patients in the total cohort felt all of the time or often that they currently had a normal life (83% [n = 38/46]) and that their disease was under control (76% [n = 35/46]; Fig 3A). Almost all patients also agreed strongly or somewhat with the statement “I feel supported by my doctor” (98% [n = 45/46]), that their doctor understood their situation (96% [n = 44/46]), and that they were well informed about their disease (91% [n = 42/46]; Fig 3B). Feeling anxious or worried about their condition was the negative statement that the highest proportion in the total cohort reported, at approximately 40% (n = 19/46; Fig 3C).

When stratified by TPO-RA use, responses to positive and negative statements were largely aligned with the total cohort. A numerically higher proportion of patients using than those not using TPO-RAs felt all the time/often that they had a normal life (89% [n = 17/19] vs 78% [n = 21/27]) and that their condition was under control (84% [n = 16/19] vs 70% [n = 19/27]). However, there was no marked difference between the cohorts regarding how informed they were about their disease (95% [n = 18/19] vs 89% [n = 24/27]), how well their doctor understood their situation (100% [n = 19/19] vs 92% [n = 25/27]), or the support they received from their doctor (100% [n = 19/19] vs 96% [n = 26/27]). For negative statements, a numerically lower proportion of patients receiving than those not receiving TPO-RAs felt worried/anxious about their condition (16% [n = 3/19] vs 59% [n = 16/27]). There was no marked difference for the other negative statements between patients using and those not using TPO-RAs.

**Fig 4:**
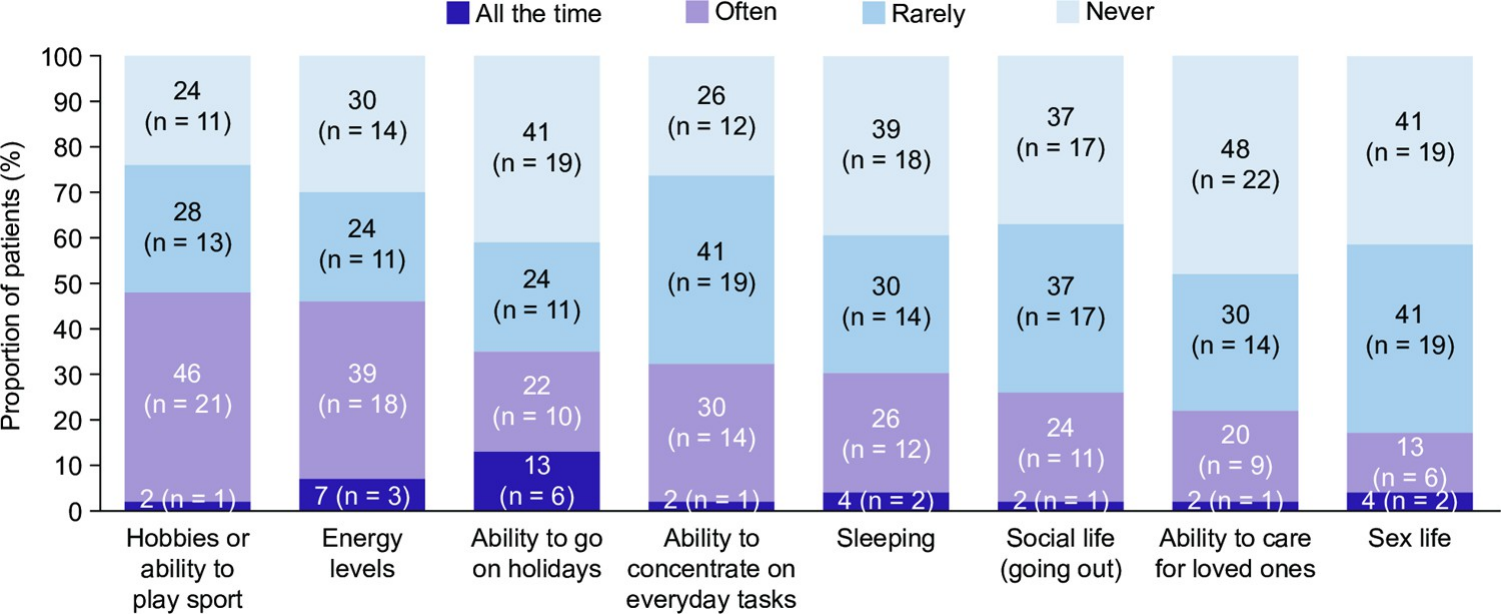
Impact of ITP on daily activities in the total cohort. Patients could select a single response per statement. Data are presented as the proportion of patients per category for each statement (N = 46).

When asked about the impact of ITP on daily activities, engagement in hobbies/ability to play sport and energy levels were most reported to be affected equally in both cohorts, with almost half of patients (48% [n = 22/46] and 46% [n = 21/46], respectively) reporting that these activities were affected all of the time or often (Fig 4).

When patients were stratified according to TPO-RA use, responses to questions about the impact of ITP on daily activities were generally aligned with the total cohort. There were some small numerical differences whether patients were receiving TPO-RAs or not for the proportion of patients for whom ITP had an impact all the time/often on sleeping (37% [n = 7/19] vs 26% [n = 7/27]), social life (going out; 21% [n = 4/19] vs 30% [n = 8/27]), or hobbies/ability to play sport (42% [n = 8/19] vs 52% [n = 14/27]).

### Impact of ITP on employment at the time of the questionnaire

In the total cohort, 72% of patients reported that they were employed. Of the total cohort, the largest proportion of patients reported that their condition had not affected their employment (63% [n = 29/46]), while 24% (n = 11/46) had considered terminating their job (but had not), 17% (n = 8/46) had changed from full-time to part-time employment, 9% (n = 4/46) had been involuntarily terminated from their job, 4% (n = 2/46) had taken early retirement, and 2% (n = 1/46) had voluntarily terminated their job owing to ITP. When asked to describe their last working week, patients from the total cohort reported that their productivity had been affected to some degree (overall mean productivity score 66.6/100).

When stratified by TPO-RA use, the impact of ITP on employment and productivity was largely aligned with the total cohort. Almost equal proportions of patients who used TPO-RA and those who did not reported that ITP had not had an impact on their employment (68% [n = 13/19] vs 59% [n = 16/27]). Shifting to part-time employment was the outcome for which there was the clearest difference according to TPO-RA use, being numerically lower in those who used TPO-RA than those who did not (11% [n = 2/19] vs 22% [n = 6/27]). For productivity, there was no difference between responses according to whether patients were using TPO-RAs or not (mean productivity scores 66.3/100 vs 66.7/100).

### Patient satisfaction with TPO-RAs

Similarly high proportions of patients were satisfied with their therapy over the previous 7 days whether they were receiving TPO-RAs (89% [n = 17/19]) or not (85% [n = 23/27]). Among patients receiving TPO-RAs, the majority were satisfied whether they were receiving TPO-RAs as a monotherapy (100% [n = 9/9]) or in combination with other therapies (80% [n = 8/10]).

When asked about their level of agreement with negative statements that relate to treatment satisfaction, a numerically higher proportion of patients receiving than those not receiving TPO-RAs agreed with the statement regarding worry about the costs of treatment (Table 2). However, patients receiving TPO-RAs were numerically less likely than those not receiving TPO-RAs to agree (strongly/somewhat) with statements regarding worry about getting infections, and wanting to stop their current treatment in the foreseeable future.

**Table 2:** ITP treatment satisfaction.

Negative statement	Using TPO-RA (n = 19)	Not using TPO-RA (n = 27)
	Agreement (strongly/somewhat), n (%)	
I am worried about the costs of my treatment	12 (63)	10 (37)
I am worried about dietary restrictions	0 (0)	6 (22)
I do not want to take the same treatment in the foreseeable future	3 (16)	10 (37)
I want to stop the treatment	3 (16)	7 (26)
I am worried about getting infections	3 (16)	6 (22)
I am worried about the physical side effects of my treatment	9 (47)	14 (52)
My treatment depletes my energy levels	6 (32)	9 (33)
I am concerned about how much time I have to take off work for administration of my treatment	6 (33)	9 (33)
My treatment is ineffective at treating my ITP symptoms	4 (21)	7 (26)
I am worried about the emotional side effects of my treatment	4 (21)	6 (22)

Abbreviations: ITP, immune thrombocytopenia; TPO-RA, thrombopoietin receptor agonist.

Patients could select a single response per statement.

## Discussion

This real-world study, which was conducted across Switzerland, Austria, and Belgium, used an online questionnaire to assess the perspective of 46 patients with primary ITP on their symptomatic burden and the impact of the disease on their lives. Importantly, whilst other studies have explored these topics using patient-reported outcomes instruments, to the best of our knowledge, none has assessed all of these topics in a single questionnaire, or assessed patient views and opinions in a similar way to the present study. This study therefore adds to the growing body of real-world evidence by providing further insight into the multifactorial burden of this rare disease on patients’ HRQoL, which goes beyond bleeding, and in some patients includes fatigue, anxiety about their condition, and limitations on their ability to engage in daily life activities, relationships, and employment [26]. To the best of our knowledge, this is also among the first real-world studies to provide insight into the treatment burden in a group of patients with ITP using TPO-RAs, and complements data collected in RCTs and extension studies.

In the present study, over 75% of patients in the total cohort experienced symptoms at diagnosis, of which the highest proportion reported bleeding. Following diagnosis, the proportion of patients experiencing symptoms was reduced to 46%, with the proportion reporting bleeding showing the greatest reduction (59% to 7%). This is encouraging and may reflect that patients received appropriate disease management following diagnosis to address their bleeding symptoms; in line with this, at the time of the questionnaire, 85% of patients were receiving treatment.

In contrast to bleeding symptoms, non-bleeding symptoms persisted at the time of the questionnaire. Fatigue in particular was reported by approximately 40% of patients both at diagnosis and at the time of the questionnaire. Results for fatigue are aligned with those of the literature that report that chronic fatigue is a commonly reported symptom in patients with ITP [38, 39]. The cause of fatigue is likely to be multifaceted, relating to inflammation in this autoimmune disorder or other poorly understood aspects of the disease, but it may also be exacerbated by the emotional strain that patients with ITP experience, such as worry/anxiety about their condition [12]. Of interest, fatigue was the symptom that the highest proportion of patients with more than one symptom wanted to resolve. This is aligned with the results of the I-WISh, and highlights the burden that fatigue presents on patients with ITP [38, 39]. Fatigue is often under-recognized by physicians, as revealed by the I-WISh [12, 38, 39]. However, the persistence of fatigue, which is independent of the type of treatment received, highlights the need for novel approaches to manage this symptom in patients with ITP.

At the time of the questionnaire, approximately 40% of patients in the total cohort felt worry/anxiety about their condition and also reported some impact of ITP on their daily life activities or employment. However, the majority of patients felt that they had a normal life, that their condition was under control, and that they were being supported by their doctor. Furthermore, at least 60% rarely/never experienced impairments in their ability to engage in daily life activities or employment. Results for impact of ITP on daily activities in the present study are largely aligned with those of the I-WISh [38, 39]. However, patients in the present study may have generally had a more positive perspective of their disease and management with regard to leading a normal life and feeling supported by their doctor than those in the I-WISh [38, 39]. This may reflect differences in the populations that were recruited into each of the studies and the ways in which patients were recruited. For example, patients in the I-WISh had a longer mean disease duration (9 vs 5 years) than those in the current study [19, 38, 39]. Furthermore, patients in the present study were recruited by haematologists and may therefore have received more active care, allowing them to feel better understood by their doctors, than patients in the I-WISh, who were recruited from patient support groups as well as by physicians [38, 39].

When comparing disease and treatment burden between patients receiving and those not receiving TPO-RAs, several outcomes did not differ according to TPO-RA use. Because the study was not powered to detect statistically significant differences, it is important not to over-interpret numerical differences between groups. However, at the time of the questionnaire, a numerically lower proportion of patients using TPO-RAs reported bleeding as a symptom, feeling worried/anxious about their condition, or shifting to part-time employment than those not using TPO-RAs, and a numerically higher proportion agreed that they had a normal life and that their condition was under control. Furthermore, although overall treatment satisfaction was similar between those using and those not using TPO-RAs, a numerically lower proportion of patients receiving TPO-RAs were worried about getting infections or wanted to stop their treatment/take a different treatment. A lower proportion of patients receiving than those not receiving TPO-RAs were also worried about dietary restrictions. Dietary restrictions are a consideration only for patients receiving certain treatments, including eltrombopag. For eltrombopag, patients need to follow strict guidelines with regard to amounts and timings of calcium intake during meals. This may indicate that a majority of patients in the cohort that received TPO-RAs were receiving romiplostim, although it was not possible from the present data to identify the specific treatments that patients were receiving. Finally, as expected, owing to the price of TPO-RAs versus some other treatments (e.g., corticosteroids), worry about cost of treatment was the only concern reported by a numerically higher proportion of patients using than those not using TPO-RAs. Further investigation is needed into the economic burden of ITP to patients from a broader perspective, including indirect costs to the patients as a result of ITP (e.g., as a result of lost wages), and their reliance on social support or other financial dependencies.

Overall, data stratified by TPO-RA use suggest that patients using TPO-RAs in the present study had a more positive perception of their clinical outcomes with regard to bleeding symptoms, and of their general psychosocial state, than those not using TPO-RAs. Results of the present study are aligned with those of clinical trials and extension studies, which have shown that TPO-RA treatment has a positive impact on patients’ HRQoL versus no TPO-RA treatment [25, 28, 29, 41]. Data in the present study are also aligned with a real-world study in which patient-reported HRQoL improved once patients switched to TPO-RAs (or between TPO-RAs) [32]. However, fatigue remained an unresolved problem in the present cohort, and needs to be more addressed in future studies.

The effectiveness and tolerability of TPO-RAs may account for their positive impact on HRQoL [42] compared with not receiving TPO-RAs. However, the improved outcomes in the cohort using TPO-RAs may also be accounted for, at least in part, by demographic and clinical differences between the cohorts. For example, a higher proportion of patients in the cohort not receiving TPO-RAs may have had newly diagnosed ITP (< 3 months since diagnosis) and may therefore have been ineligible to receive this treatment. Patients who are newly diagnosed may also be more likely to have a negative perception of their condition because they have not yet had time to come to terms with their diagnosis, or because they are not yet receiving appropriate management that can reduce symptoms (e.g., bleeding) that have an impact on their HRQoL. Indeed, among patients who were not receiving TPO-RAs, 26% were not receiving any treatment at the time of the questionnaire. Owing to the design of the questionnaire patients could be classified only according to whether they had chronic disease or not, and so it was not possible to assess how many patients in the study had acute disease.

### Study limitations

One of the main limitations of this study is that patient recruitment was not monitored. During the invitation to participate, it was explicitly emphasized to the patient that there would be no way to identify whether they had participated or not to ensure that those who did respond could provide their opinions anonymously, so that they could respond honestly about sensitive topics such as medical care satisfaction and about their personal lives. It was therefore not possible to investigate how many patients were contacted for participation. Furthermore, barriers to completing the questionnaire, which may have resulted in non-participation, were also not investigated. Finally, there was no requirement or incentive for physicians to meet patient recruitment quotas; it was entirely up to physicians whether they wanted to hand out envelopes with unique access codes to the questionnaire to their patients. These factors, in combination with ITP being a rare disease, may account for the rather low number of participants (N = 46) recruited during the active periods of the study (3 periods over 3 months), over 2 years. Nevertheless, this study provides important insight into the views and feelings of a cohort of patients with ITP, and the impact of ITP on their lives, including in those receiving TPO-RAs.

The questionnaire used closed questions and all responses were anonymous. It was therefore not possible to investigate outcomes further, or to correlate them with clinical characteristics that were not collected as part of the questionnaire (e.g., platelet counts). Because responses also relied on patients’ recollection of symptoms and treatment received at diagnosis, it is possible that they were not an accurate reflection of patients’ experiences at that time, particularly for those who were diagnosed several years before completing the questionnaire. The online nature of the questionnaire may have also precluded some patients from participating in the study, and may account for the numerically lower numbers of patients recruited who were over 60 years of age (24%) than who were in the younger age groups.

Another limitation of the questionnaire is that it was not validated. However, the topics included in this questionnaire were selected by a panel of haematologists with extensive experience in, and dedication to, caring for patients with ITP. Therefore, the questionnaire is likely to reflect issues that impact patients in their daily lives and are of importance to them. Furthermore, questions were phrased in a way that was consistent with the language used by haematologists when discussing with patients about their physical and emotional condition and their HRQoL. Therefore, it was agreed by the panel of haematologists that the questionnaire was suitable for use in the present study.

Finally, patients recruited into the study were being treated by haematologists. They may therefore have had more severe disease or required treatment, compared with patients managed by general practitioners or who were not receiving or in need of treatment or management by a healthcare professional, who would not have been represented in the study. Furthermore, physicians who sought to recruit patients to participate in the study may have also actively engaged in enquiring about their patients’ experiences and keeping patients informed of their condition; this would have had a positive impact on their patients’ lives and may have produced biased questionnaire results. Based on these limitations, the results of this study are not generalizable to the general population of patients with primary ITP, but they do provide valuable data from the patient’s perspective in a selected group of patients with primary ITP. To overcome some of these limitations, a future study could recruit patients with ITP from other sources, such as patient registries (e.g. the German ITP registry, which is currently being established), that may include a broad population of patients, including those who are not under the care of haematologists or any healthcare professional.

## Conclusion

There is growing recognition of the importance of evaluating patients’ perspectives of the impact of their disease and treatment on their lives. The current study, which was conducted across Switzerland, Austria, and Belgium, provides further insight into the burden of ITP based on the feelings and opinions expressed by a cohort of patients in clinical practice. This is particularly relevant with regard to fatigue, the impact of which is often underestimated, and which is not generally responsive to available treatments. Importantly, to the best of our knowledge, this is also among the first studies assessing the patient-reported experience in those receiving versus those not receiving TPO-RAs: patients receiving TPO-RAs had better outcomes with regard to bleeding, but also for other aspects of the disease, such as anxiety.

## Supporting information

S1 File Immune Thrombocytopenia (ITP) Patient survey questionnaire. (DOCX)
