# Supplementary material for: Real-world impact of primary immune thrombocytopenia and treatment with thrombopoietin receptor agonists on quality of life based on patient-reported experience: Results from a questionnaire conducted in Switzerland, Austria, and Belgium
Source: PLoS One. 2022 Apr 21;17(4):e0267342. doi: 10.1371/journal.pone.0267342 (PMC9022837; doi:10.1371/journal.pone.0267342)
Supplement: S1 File — (DOCX) [file pone.0267342.s001.docx]

**Supporting Information: Questionnaire**

Thank you for your interest in participating in the Immune Thrombocytopenia (ITP) Patient survey. Here is some important information for you:

- **The aim of this survey is to learn from affected patients what kind of burdens an ITP disease can bring.** It is hoped that the results of the survey will help to develop targeted assistance for the problems caused by the disease.
- **Your privacy is protected.** Your individual replies will remain **anonymous** and will not be shared with your doctor or anyone else.
- Your help and participation in this survey will not result in any change in your treatment, nor will it alter the relationship with your doctor or the quality of care received.

The questionnaire was developed by 4 Swiss specialists for this disease: Dr. Alicia Rovó (Inselspital Bern), Dr. Kaveh Samii (HUG, Hôpitaux Universitaires de Genève), Dr. Nathan Cantoni (Kantonsspital Aarau) and Dr. Rudolf Benz (Kantonsspital Münsterlingen) with the participation of Novartis Pharma Schweiz AG. Polyquest - a Swiss market research agency in Bern - has been commissioned to program and conduct the online survey. Polyquest will also evaluate the answers.

With your participation …

- … you grant permission for the researching agency (Polyquest) to pass the summarized results of the survey (not your personal answers, but only summarized results) to the working group of the physicians involved, to Novartis and to persons/companies cooperating with the working group or Novartis in this context.
- … you grant permission for the results (not your personal answers, but only summarized results) to be published.

| Button | The following text only appears if the respondent clicks the button. |
| --- | --- |
| Label of the button: | What happens when I fill out the questionnaire and what happens to my answers afterwards? |

It is up to you whether you participate or not. You can discontinue answering the questions at any time without giving any reasons. **If you do not participate, you will not incur any disadvantages**.

If you choose to participate, we ask you to answer the questions alone and without the help of your doctor. **It takes about 15 minutes to complete the questionnaire**.

**I confirm that I have read and understood the above information. I am aware that I do not have to participate and that I can cancel the completion of the questionnaire at any time.**

**If you would like to participate, please give your consent here by answering 'Agree'.**

| ⭘ | Agree |
| --- | --- |
| ⭘ | Disagree -> NO INTERVIEW |

| Logic | Screen out if code 2 ‘disagree’ |
| --- | --- |

PATIENT SCREENER

S1. I confirm that I have ITP (Immune thrombocytopenia/Idiopathic thrombocytopenia) <ASK ALL>

Please select one answer

| ⭘ | Yes |
| --- | --- |
| ⭘ | No |

| Logic | If ‘No’ selected – screen out |
| --- | --- |

S2. How old are you? <ASK ALL>

Please enter your age in the box below

| ________ years (age) |
| --- |

| Logic | If <18 – screen out / Programming: Only input of numbers allowed! |
| --- | --- |

S3. I'm living in <ASK ALL>

| ⭘ | Switzerland |
| --- | --- |
| ⭘ | Austria |
| ⭘ | Belgium |
| ⭘ | Somewhere else –> screen out |

SECTION A: DIAGNOSIS

Q1. Please specify your gender. <ASK ALL>

| ⭘ | Male |
| --- | --- |
| ⭘ | Female |

Q2. What age were you when you were formally diagnosed with ITP? <ASK ALL>

| ________ years (age) |
| --- |

Q3. To the best of your knowledge, how many physicians did you see before receiving your formal ITP diagnosis? <ASK ALL>

| ________ number of physicians seen |
| --- |

SECTION B: SYMPTOMS

Q1. How would you describe your current state of health? <ASK ALL>

Please click on the bar below. Clicking next to the green smiley means excellent health, whereas clicking next to the red smiley means very poor health.

You need to move the slider! If you want to leave the slider in the initial position, you must move it briefly and then slide it back.


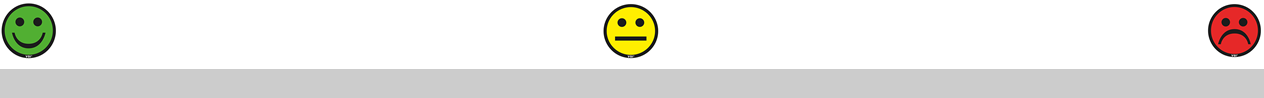


Q2. Which of the following symptoms did you have at the time of ITP diagnosis? <ASK ALL>

Please select all that apply

| 🞎 | Bleeding |
| --- | --- |
| 🞎 | Thrombosis – formation of a blood clot within a blood vessel |
| 🞎 | Fatigue (tiredness) |
| 🞎 | Headaches |
| 🞎 | Dizziness |
| 🞎 | Sleep deprivation |
| 🞎 | No symptoms |

<IF BLEEDING IS SELECTED, SHOW THE FOLLOWING>

Q2b. You have indicated bleeding. Can you please specify the type of bleeding at the time of ITP diagnosis?

| 🞎 | Purpura  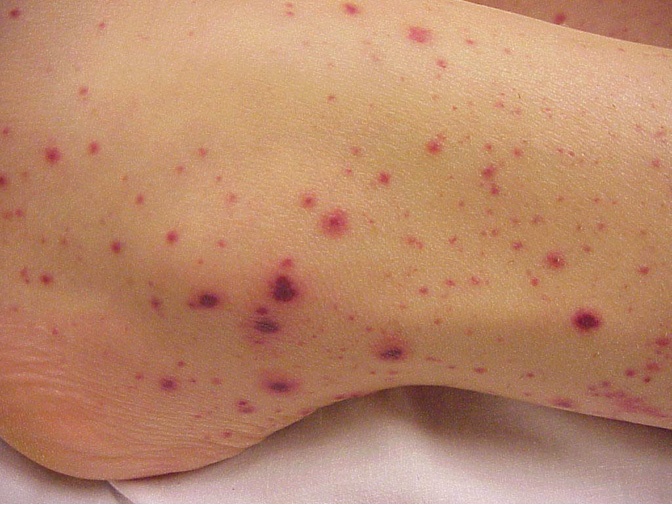 |
| --- | --- |
| 🞎 | Petechiae  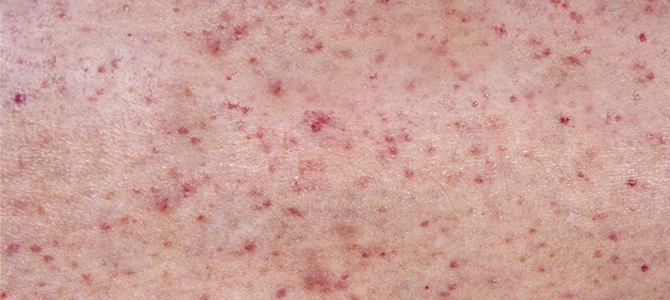 |
| 🞎 | Hematoma  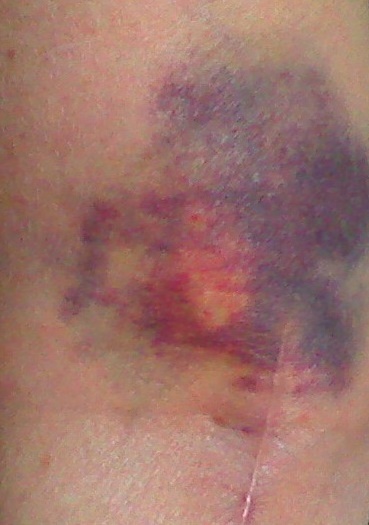 |
| 🞎 | Nosebleeds |
| 🞎 | Bleeding from the gums (e.g. during dental work) |
| 🞎 | Prolonged bleeding from cuts |
| 🞎 | Internal bleeding (organ bleeding) |
| 🞎 | Blood in the urine or stool (bowel movement) |
| 🞎 | Heavy menstrual bleeding |

Q3. Which of the following symptoms do you have today? <ASK ALL>

Please select all that apply

| 🞎 | Bleeding |
| --- | --- |
| 🞎 | Thrombosis – formation of a blood clot within a blood vessel. |
| 🞎 | Fatigue (tiredness) |
| 🞎 | Headaches |
| 🞎 | Dizziness |
| 🞎 | Sleep deprivation |
| 🞎 | No symptoms |

<IF BLEEDING IS SELECTED, SHOW THE FOLLOWING>

Q3b. You have indicated bleeding. Can you please specify the type of bleeding?

| 🞎 | Purpura  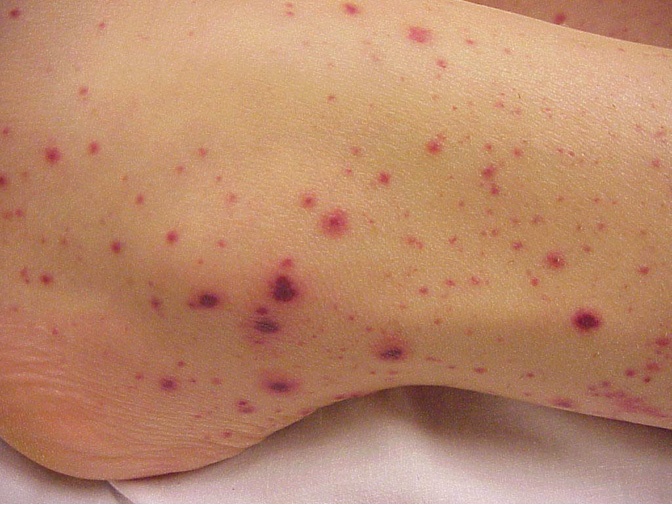 |
| --- | --- |
| 🞎 | Petechiae  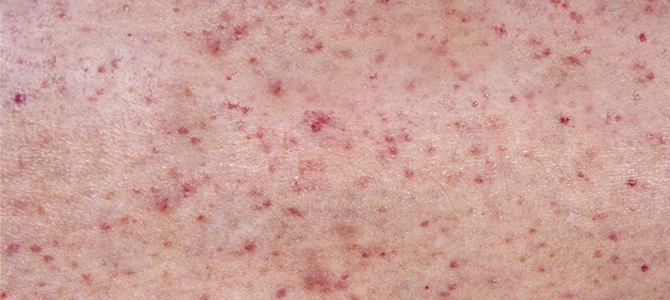 |
| 🞎 | Hematoma  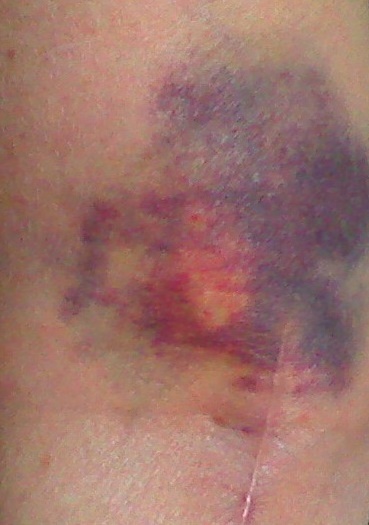 |
| 🞎 | Nosebleeds |
| 🞎 | Bleeding from the gums (e.g. during dental work) |
| 🞎 | Prolonged bleeding from cuts |
| 🞎 | Internal bleeding (organ bleeding) |
| 🞎 | Blood in the urine or stool (bowel movement) |
| 🞎 | Heavy menstrual bleeding |

Q4. Please rate the severity of the symptoms you have today. <ONLY THE SYMPTOMS THAT HAVE BEEN SELECTED IN Q3 ARE SHOWN>

Please rate each symptom below by clicking on the corresponding bar. Clicking next to the green smiley means not severe at all, whereas clicking next to the red smiley means very severe.

You need to move each slider! If you want to leave the slider in the initial position, you must move it briefly and then slide it back.


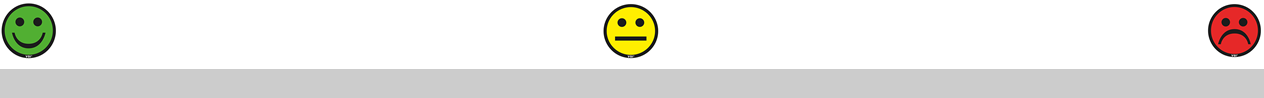


| 🞎 | Purpura  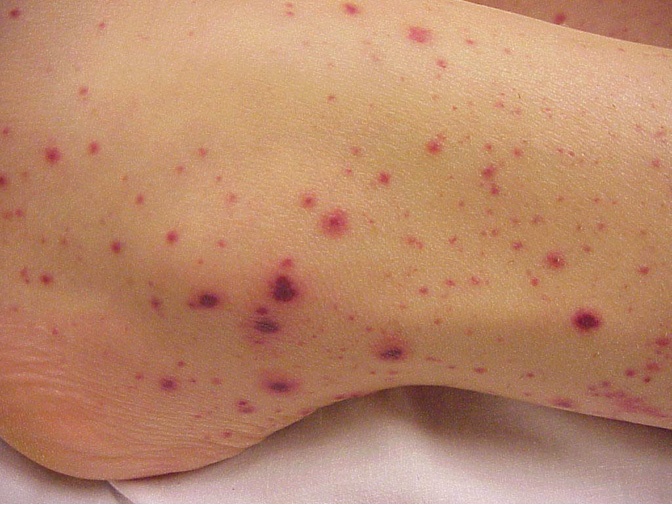 |
| --- | --- |
| 🞎 | Petechiae  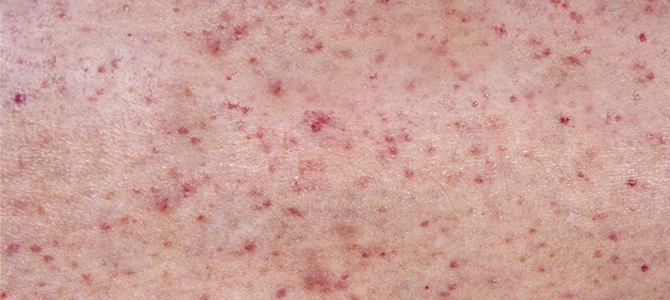 |
| 🞎 | Hematoma  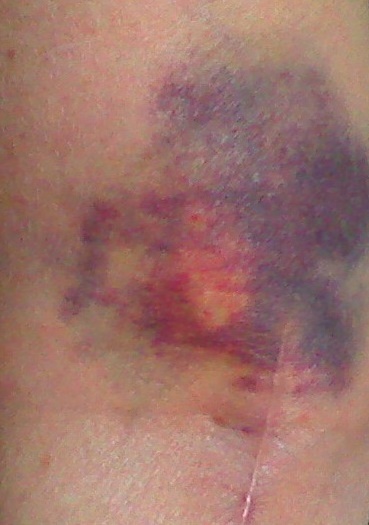 |
| 🞎 | Nosebleeds |
| 🞎 | Bleeding from the gums (e.g. during dental work) |
| 🞎 | Prolonged bleeding from cuts |
| 🞎 | Internal bleeding (organ bleeding) |
| 🞎 | Blood in the urine or stool (bowel movement) |
| 🞎 | Heavy menstrual bleeding |
| 🞎 | Thrombosis – formation of a blood clot within a blood vessel |
| 🞎 | Fatigue (tiredness) |
| 🞎 | Headaches |
| 🞎 | Dizziness |
| 🞎 | Sleep deprivation |
| 🞎 | No symptoms |

Q5. Which symptom that you have today would you most want to resolve? <ONLY THE SYMPTOMS THAT HAVE BEEN SELECTED IN Q3 ARE SHOWN. IF ≥4 SYMPTOMS WERE SELECTED, THE FIRST ONE THAT IS CLICKED DISAPPEARS AND THE QUESTION IS ASKED AGAIN (3 SYMPTOMS TO BE CLICKED IN TOTAL). IF 3 SYMPTOMS WERE SELECTED, THE QUESTION IS ASKED ONLY ONCE>

| 🞎 | Purpura  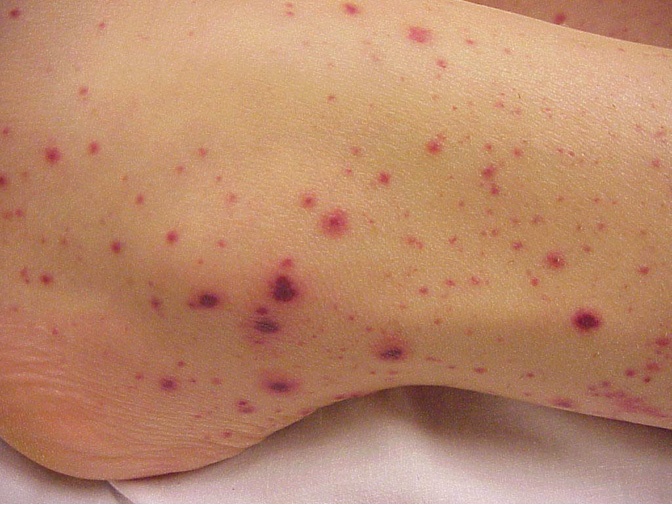 |
| --- | --- |
| 🞎 | Petechiae  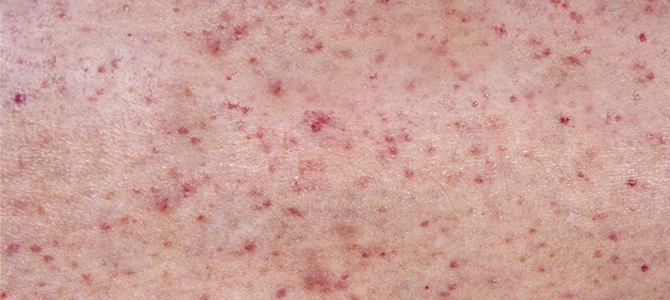 |
| 🞎 | Hematoma  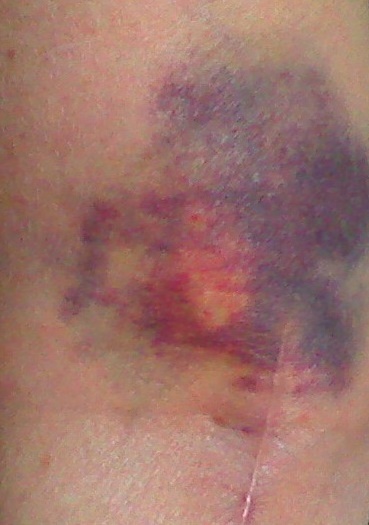 |
| 🞎 | Nosebleeds |
| 🞎 | Bleeding from the gums (e.g. during dental work) |
| 🞎 | Prolonged bleeding from cuts |
| 🞎 | Internal bleeding (organ bleeding) |
| 🞎 | Blood in the urine or stool (bowel movement) |
| 🞎 | Heavy menstrual bleeding |
| 🞎 | Thrombosis – formation of a blood clot within a blood vessel |
| 🞎 | Fatigue (tiredness) |
| 🞎 | Headaches |
| 🞎 | Dizziness |
| 🞎 | Sleep deprivation |

If we look now at the list of remaining symptoms: Which symptom that you have today would you most want to resolve from this remaining list?

If we look now at the list of remaining symptoms: Which symptom that you have today would you most want to resolve from this remaining list?

SECTION C: IMPACT ON DAILY lIFE

Q1. Does your ITP impact your … <ASK ALL>

|  | **Never** | **Rarely** | **Often** | **All the time** |
| --- | --- | --- | --- | --- |
| …ability to concentrate on everyday tasks? | ⭘ | ⭘ | ⭘ | ⭘ |
| …social life (going out)? | ⭘ | ⭘ | ⭘ | ⭘ |
| …sex life? | ⭘ | ⭘ | ⭘ | ⭘ |
| …energy levels? | ⭘ | ⭘ | ⭘ | ⭘ |
| …ability to care for loved ones? | ⭘ | ⭘ | ⭘ | ⭘ |
| …hobbies or ability to play sport? | ⭘ | ⭘ | ⭘ | ⭘ |
| …ability to go on holidays? | ⭘ | ⭘ | ⭘ | ⭘ |
| …sleeping? | ⭘ | ⭘ | ⭘ | ⭘ |

| <ASK ALL> | **Yes** | **No** |
| --- | --- | --- |
| Q2. Has your ITP impacted your pregnancy plans? | ⭘ | ⭘ |

SECTION D: EMOTIONAL BURDEN

Please tick one box.

Q1. How often do you feel as described in these positive statements here? <ASK ALL>

|  | **Never** | **Rarely** | **Often** | **All the time** |
| --- | --- | --- | --- | --- |
| I have a normal life | ⭘ | ⭘ | ⭘ | ⭘ |
| I feel that my condition is under control | ⭘ | ⭘ | ⭘ | ⭘ |
| I forget that I have a disease | ⭘ | ⭘ | ⭘ | ⭘ |

Q2. How often do you feel as described in these negative statements here? <ASK ALL>

|  | **Never** | **Rarely** | **Often** | **All the time** |
| --- | --- | --- | --- | --- |
| My condition is controlling my life | ⭘ | ⭘ | ⭘ | ⭘ |
| I feel sad about my condition | ⭘ | ⭘ | ⭘ | ⭘ |
| I feel depressed about my condition | ⭘ | ⭘ | ⭘ | ⭘ |
| I feel anxious/worried about my condition | ⭘ | ⭘ | ⭘ | ⭘ |
| My condition causes emotional hardship for me | ⭘ | ⭘ | ⭘ | ⭘ |

SECTION E: IMPACT ON WORK, FINANCES AND USE OF CAREGIVER

Q1. Are you currently employed (working for pay) or self-employed? <ASK ALL>

| ⭘ | Yes |
| --- | --- |
| ⭘ | No |

Q2. As a result of your ITP, have you ever… <ASK ALL>

Please select all that apply

|  | **Yes** | **No** |
| --- | --- | --- |
| Changed from full-time to part-time employment? | ⭘ | ⭘ |
| Taken early retirement? | ⭘ | ⭘ |
| Voluntarily terminated your job? | ⭘ | ⭘ |
| Been involuntarily terminated from your job | ⭘ | ⭘ |
| Considered terminating your job (but did not terminate for reasons such as health insurance coverage, etc.)? | ⭘ | ⭘ |

Q3. (WPAI Q2) During the past seven days, how many hours did you miss from work because of problems associated with your ITP condition? *Include hours you missed on sick days, times you went in late, left early, etc. because of your ITP, time spent going to the doctor because of your ITP.*

| ________ Hours |
| --- |

| Range | 0-168 |
| --- | --- |

Q3a. How would you describe the last week in this respect? Was this more like... <ASK ALL>

| ⭘ | a normal week |
| --- | --- |
| ⭘ | a week that was better than usual |
| ⭘ | a week that was worse than usual |

Q4. During the past 7 days, how much did your ITP condition affect your productivity while you were working?

**Please click on the following. Clicking next to the green smiley means that ITP had no effect on your work, whereas clicking next to the red smiley means ITP completely prevented you from working.**

**You need to move each slider! If you want to leave the slider in the initial position, you must move it briefly and then slide it back.** <ASK IF SECTION D Q1 IS 'YES'>


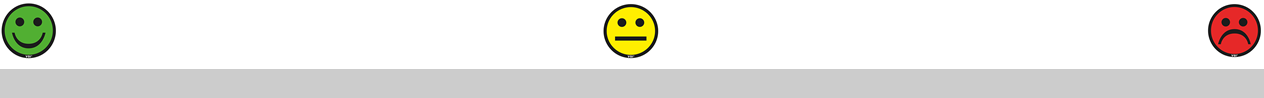


Q5. How often do you rely on someone (i.e. a caregiver, family member, friend or neighbour) to assist you with your activities of daily living due to your ITP? <ASK ALL>

*Assistance with activities of daily living can range from a few hours of shopping and cleaning to intensive medical or personal care. Tasks can include shopping, house cleaning, cooking, giving medications, toileting assistance and so forth. The person who assists you with your daily activities will be referred to as “****caregiver****” in the following survey.*

Please select one answer

| ⭘ | Never |
| --- | --- |
| ⭘ | Rarely |
| ⭘ | Often |
| ⭘ | All the time |

Q6. Who is your main caregiver? <ASK IF SECTION D Q5 IS 'RARELY', 'OFTEN', 'ALL THE TIME'>

Please select one answer

| ⭘ | Spouse/partner |
| --- | --- |
| ⭘ | Son/daughter |
| ⭘ | Parent |
| ⭘ | Sibling |
| ⭘ | Other relative |
| ⭘ | Friend |
| ⭘ | Neighbour |
| ⭘ | Professional caregiver |

Q7. What kind of help do you require from your main caregiver as a result of your ITP condition?

<ASK IF SECTION D Q5 IS 'RARELY', 'OFTEN', 'ALL THE TIME'>

Please select all that apply

| 🞎 | Companionship (e.g. talking, reading, keeping company), emotional support or encouragement |
| --- | --- |
| 🞎 | Transportation (e.g. driving to doctor’s appointments, driving for errands) |
| 🞎 | Homemaking (e.g. shopping, cleaning, preparing meals) |
| 🞎 | Personal care assistance (e.g. feeding, bathing, toileting, dressing, grooming) |
| 🞎 | Healthcare assistance (e.g. help with medications, wound care, researching condition) |
| 🞎 | Managing finances (e.g. paying bills, managing budget) |
| 🞎 | Help plan and organize everyday activities |

SECTION E: TREATMENTS

Q1. Please select all treatments you are receiving today to help manage your ITP <ASK ALL>

Please select all that apply

| 🞎 | **Corticosteroids** (Dexagenta, Dexamethason, Fortecortin, Lodotra, Methylprednison, Medrol, Mephameson, Prednison, Spiricort) |
| --- | --- |
| 🞎 | **Androgens** (Andriol , Nebido, Testosteron, Testoviron) |
| 🞎 | **Cyclosporin** (Ciqorin, Ikervis, Sandimmun) |
| 🞎 | **Rituximab** (Mabthera) |
| 🞎 | **Anti-fibrinolytics** (Ciklokapron, Tranexam, Trasylol) |
| 🞎 | **Azathioprine** (Azafalk, Azaimun, Azarek, Imurek) |
| 🞎 | **Cyclophosphamide** (Endoxan) |
| 🞎 | **Dapsone** |
| 🞎 | **Intravenous Immunoglobin** |
| 🞎 | **Mycophenolic acid** (Myfortic) |
| 🞎 | **Platelet transfusion** |
| 🞎 | **Splenectomy** |
| 🞎 | **Alternative medicine** (i.e. homoeopathy) |
| 🞎 | **Thrombopoietin receptor agonists** (Nplate, Revolade) |
| 🞎 | **Other** |
|  |  |
| ⭘ | **No therapy** |

Q2. Please select all treatments you have **ever** received to help manage your ITP that you can still remember.

<ASK ALL>

Please select all that apply. Current therapies have already been selected.

| 🞎 | **Corticosteroids** (Dexagenta, Dexamethason, Fortecortin, Lodotra, Methylprednison, Medrol, Mephameson, Prednison, Spiricort) |
| --- | --- |
| 🞎 | **Androgens** (Andriol , Nebido, Testosteron, Testoviron) |
| 🞎 | **Cyclosporin** (Ciqorin, Ikervis, Sandimmun) |
| 🞎 | **Rituximab** (Mabthera) |
| 🞎 | **Anti-fibrinolytics** (Ciklokapron, Tranexam, Trasylol) |
| 🞎 | **Azathioprine** (Azafalk, Azaimun, Azarek, Imurek) |
| 🞎 | **Cyclophosphamide** (Endoxan) |
| 🞎 | **Dapsone** |
| 🞎 | **Intravenous Immunoglobin** |
| 🞎 | **Mycophenolic acid** (Myfortic) |
| 🞎 | **Platelet transfusion** |
| 🞎 | **Splenectomy** |
| 🞎 | **Alternative medicine** (i.e. homoeopathy) |
| 🞎 | **Thrombopoietin receptor agonists** (Nplate, Revolade) |
| 🞎 | **Other** |
|  |  |
| ⭘ | **No therapy** |

Please select all that apply

Q3. How long have you been receiving your current treatment? <ASK ALL>

Please select the relevant unit below

| ⭘ Weeks | ⭘ Months | ⭘ Years | ⭘ Don’t know |
| --- | --- | --- | --- |

Q4. Thinking about the last 7 days, overall how satisfied have you been with your therapy for your ITP condition?

You need to move each slider! If you want to leave the slider in the initial position, you must move it briefly and then slide it back. <ASK ALL>


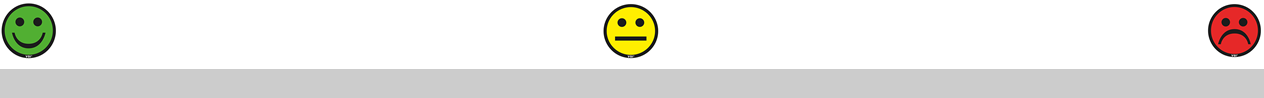


Q5. How much do you agree with the following statements in relation to the treatment you are currently getting for your ITP condition?

|  | **strongly agree** | **somewhat agree** | **somewhat disagree** | **strongly disagree** |
| --- | --- | --- | --- | --- |
| My treatment is ineffective at treating my ITP symptoms | ⭘ | ⭘ | ⭘ | ⭘ |
| My treatment depletes my energy levels | ⭘ | ⭘ | ⭘ | ⭘ |
| I am worried about the physical side effects of my treatment | ⭘ | ⭘ | ⭘ | ⭘ |
| I am worried about the emotional side effects of my treatment | ⭘ | ⭘ | ⭘ | ⭘ |
| I am worried about getting infections | ⭘ | ⭘ | ⭘ | ⭘ |
| I am worried about dietary restrictions | ⭘ | ⭘ | ⭘ | ⭘ |
| I want to stop the treatment | ⭘ | ⭘ | ⭘ | ⭘ |
| I am worried about the costs of my treatment | ⭘ | ⭘ | ⭘ | ⭘ |
| I do not want to take the same treatment in the foreseeable future | ⭘ | ⭘ | ⭘ | ⭘ |
| I am concerned about how much time I have to take off work for administration of my treatment | ⭘ | ⭘ | ⭘ | ⭘ |

Q6. How much do you agree with the following statements?

|  | **strongly agree** | **somewhat agree** | **somewhat disagree** | **strongly disagree** |
| --- | --- | --- | --- | --- |
| I am well informed about my disease | ⭘ | ⭘ | ⭘ | ⭘ |
| I feel supported by my doctor | ⭘ | ⭘ | ⭘ | ⭘ |
| My doctor understands my situation | ⭘ | ⭘ | ⭘ | ⭘ |

That's the end of the questionnaire. Thank you very much for your time and your commitment to answer our questions.
